# Temporal coherence effects on voice attribution in multi-speaker stream segregation

**DOI:** 10.1121/10.0036672

**Published:** 2025-05-08

**Authors:** Jaeeun Lee, Andrew J. Oxenham

**Affiliations:** Department of Psychology, University of Minnesota, Minneapolis, Minnesota 55455, USA lee03881@umn.edu, oxenham@umn.edu

## Abstract

The principle of temporal coherence predicts that two temporally coherent voices should form a unified auditory stream, whereas incoherent voices should form separate streams. This prediction was tested by asking 20 normal-hearing listeners to identify the last word spoken by the higher or lower of two talkers, preceded by temporally coherent or incoherent phrases spoken by the same two talkers, or by silence. In contrast to results from stream-segregation studies using simple repeating stimuli that manipulated temporal coherence, no significant differences in performance were observed between the conditions, raising questions regarding the generalization of temporal-coherence principles to complex speech.

## 1. Introduction

The human auditory system relies on various acoustic cues to integrate information originating from the same source, or auditory object, while simultaneously segregating information from other sources (Bregman, 1994; Carlyon, 2004; McDermott, 2009; Middlebrooks *et al.*, 2017). For speech, the question of how this process is achieved has been termed the cocktail party problem (Cherry, 1953). A prominent cue for perceptually grouping different features or frequency components is temporal coherence (Elhilali *et al.*, 2009; Shamma *et al.*, 2011). The principle of temporal coherence states that sounds with synchronized timing are typically perceived as coming from a single source, while those that are temporally incoherent are more likely to be interpreted as originating from different sources.

Demonstrations of the perceptual effects of temporal coherence have typically used tasks involving the judgment of a single target sound, with the temporal coherence of the preceding sound environment manipulated to affect the perception of the target. For instance, Elhilali *et al.* (2009) studied asynchrony detection between two pure tones of different frequencies. When the tones were presented in isolation or presented following a sequence of temporally coherent pair of tones at the same frequency as the target tones, listeners were highly sensitive to any asynchrony between the target tones, achieving asynchrony detection thresholds of 2–4 ms. In contrast, when the same target tones were preceded by a sequence of the same tones, but presented incoherently, listeners found it much more difficult to detect an asynchrony, suggesting that they were perceived in different streams.

Another example was provided by Krogholt Christiansen and Oxenham (2014). They used comodulation masking release (e.g., Hall *et al.*, 1990), with a target tone embedded in a narrowband noise masker and spectrally flanking narrowband noisebands that were either uncorrelated or comodulated with the on-frequency masker noiseband. Previous work had shown that comodulation masking release (the difference in masked threshold between comodulated and independent flanker bands) depended on the flanking bands being perceptually grouped with the on-frequency masker band (Dau *et al.*, 2009; Grose and Hall, 1993). To examine the effect of temporal coherence, Krogholt Christiansen and Oxenham (2014) preceded the simultaneous masker and flanking bands with sequences of masker and flankers that were either coherent (all bands were gated on and off synchronously) or incoherent (masker and flankers followed regular, but different, temporal patterns). They found that comodulation masking release was abolished when the preceding maskers and flankers were incoherent, in line with predictions based on the perceptual segregation of the temporally incoherent bands.

More recently, Sollini *et al.* (2022) found that the detection of a tone interleaved within an otherwise brief regular sequence of tones at the same frequency was facilitated both by temporal regularity and temporal coherence of the preceding tone environment.

So far, the importance of temporal coherence has been demonstrated primarily by using simple repeating sequences of psychoacoustic stimuli. Although a recent study combining single-unit cortical recordings in ferret and computational modeling highlighted the potential role of temporal coherence in the segregation of competing speech (Joshi *et al.*, 2024), no behavioral studies have yet provided experimental demonstrations of how temporal coherence affects speech segregation.

Indeed, a recent study found little evidence for the importance of temporal coherence in segregating two competing voices, when introducing temporal asynchrony between frequency bands (Lee and Oxenham, 2024). The present study used a different approach, analogous to the psychoacoustic experiments of Elhilali *et al.* (2009) and Krogholt Christiansen and Oxenham (2014), whereby a final pair of simultaneous words were preceded by phrases that were either temporally coherent or incoherent. Our prediction was that the target words would be easier to segregate and identify if preceded by temporally incoherent phrases, encouraging segregation, than if they were preceded by temporally coherent phrases, encouraging grouping, or silence.

## 2. Methods

### 2.1 Listeners

Twenty normal-hearing listeners participated in this study. The group consisted of native speakers of American English aged between 19 and 41 years (mean = 22 years; 16 females, 4 males) with pure-tone audiometric thresholds no greater than 20 dB HL at octave frequencies from 250 Hz to 8 kHz. All participants were recruited through the University of Minnesota Psychology research participant pool and were compensated with their choice of extra course credit or hourly payment. They completed the experiment within a single session, which lasted around 1 hour, including breaks. The protocol was approved by the University of Minnesota's Institutional Review Board, and the participants provided written informed consent.

### 2.2 Stimuli

The speech stimuli used in the experiment were recorded by an American English-speaking female in a sound-attenuating booth located in a quiet room. All sounds were recorded at a 48-kHz sampling rate with 24-bit resolution using an ME64 stationary microphone (Sennheiser) connected to a Zoom H4n Pro recorder. Two different precursor phrases were recorded (“The last word you will hear is…” and “You will also hear the word…”), along with three consonant-vowel-consonant target words (BAT, BEET, and BOOT). The mean F0 of the precursor phrases was 200 Hz, and the mean F0 of the three words was 203, 209, and 197 Hz for BAT, BEET, and BOOT, respectively. For the experiment, the F0s of the original recordings were shifted up and down by 1.5 semitones using PRAAT (version 6.3) (Boersma and Weenick, 2024) to generate a “higher” voice and a “lower” voice, separated by 3 semitones. The duration of the sentence (phrase and target word, e.g., “You will also hear the word bat”) was approximately 2 s. As noted by Sollini *et al.* (2022), a duration of between 0.4 and 2.5 s has been shown to produce maximal effect of buildup in stream segregation, depending on the task and stimuli, so our sentence duration of about 2 s should be sufficient to induce segregation between incoherent streams. The level of each voice was set to 65 dB SPL, yielding a target-to-masker ratio of 0 dB.

**Fig. 1.**
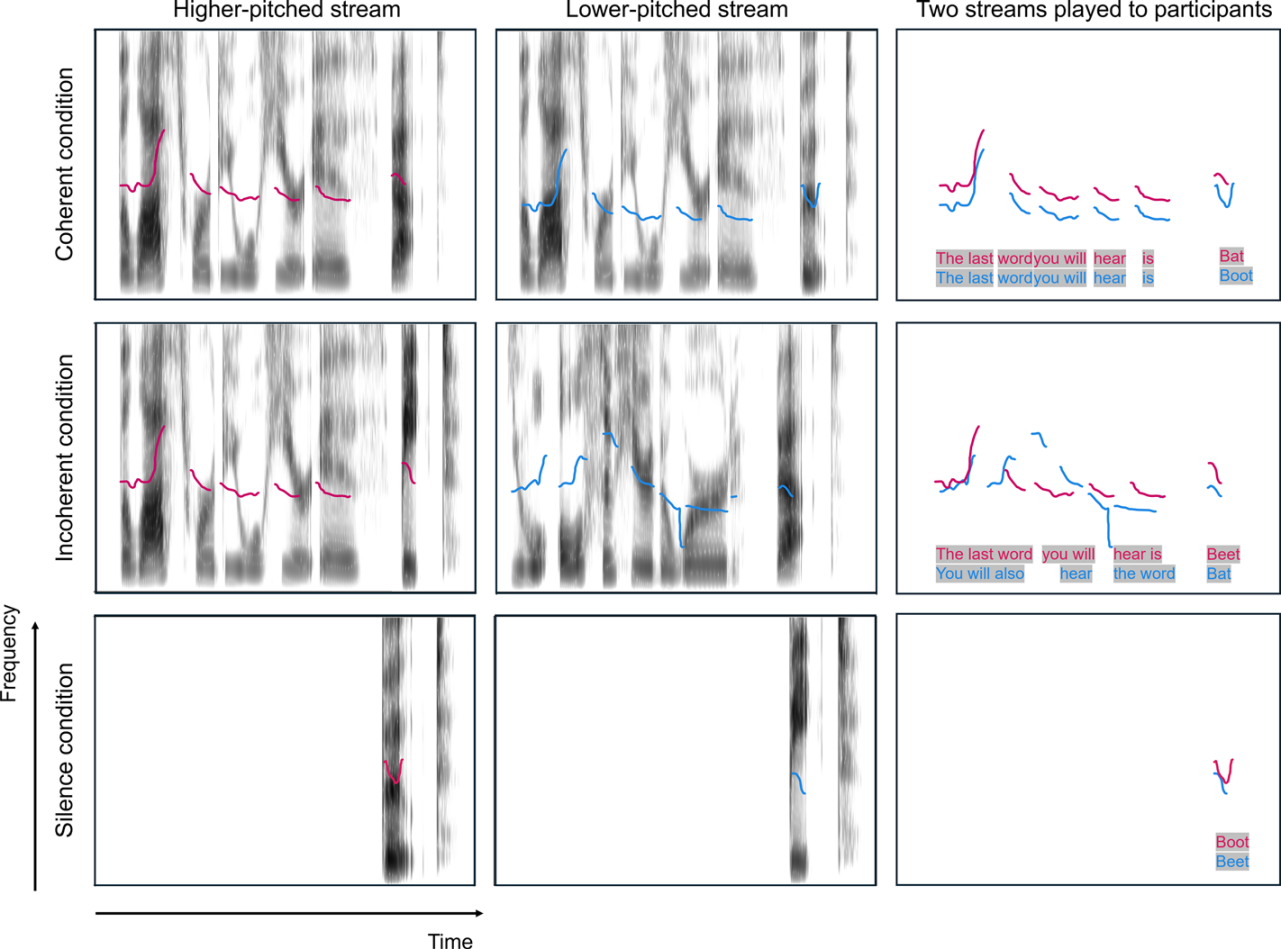
Example stimuli of three different precursor conditions. Sample stimuli for each condition are available: Mm. 1, Mm. 2, Mm. 3. The magenta pitch contour shows the higher voice. The blue pitch contour shows the lower voice.

Three conditions were tested: “coherent,” “incoherent,” and “silence” (Mm. 1, Mm. 2, and Mm. 3) In the coherent condition, the two different-pitched voices spoke the same precursor phrase (selected at random on each trial) synchronously, followed immediately by two different target words. In the incoherent condition, the two voices spoke different phrases (with the two target phrases randomly assigned to high and low voice), also followed by two different target words. In the silence condition, a 2-s silent segment preceded the two different target words. In all three conditions, the target words were presented synchronously (Fig. 1).

**Mm. 1.** Coherent condition.

**Mm. 2.** Incoherent condition.

**Mm. 3.** Silence condition.

### 2.3 Procedure

The experiment was blocked according to the stimulus condition and attended voice pitch. Each block had 12 trials, and all the combinations of three conditions (coherent, incoherent, or silence) and two instructions (attend high or low) were repeated 5 times, resulting in a total of 30 blocks and 360 trials. Listeners were given a text prompt at the beginning of each trial which instructed them to attend to either the higher or the lower voice. Their task was to report which target word was spoken by the attended voice (BAT, BEET, or BOOT) using the keys 1, 2, and 3 on the keyboard. The listeners completed six practice trials that included two examples of each condition (one attending to the high voice and one to the low voice) before embarking on the main experiment. Feedback was provided during the practice trials to help listeners understand the task, but none was given during the main trials. All stimuli were presented to listeners diotically over HD650 headphones (Sennheiser, Old Lyme, CT) via a Lynx E22 sound card (Lynx Studio Technologies, Costa Mesa, CA) at a sampling rate of 48 kHz and resolution of 24 bits in a sound-attenuating booth. Listeners completed the experiment via a graphical user interface, generated using Psychophysics Toolbox extensions (Brainard and Vision, 1997; Kleiner *et al.*, 2007) in MATLAB (The MathWorks, 2022).

### 2.4 Analysis

To quantify the temporal coherence of the stimuli, we calculated the cross-covariance (Pearson's product-moment correlation) of the temporal envelopes between the two precursor phrases. As expected for the broadband envelopes (lowpass filtered at 50 Hz to remove F0 periodicity), the low and high versions of the same precursor phrases had a cross-covariance value near 1, indicating that they were temporally coherent, whereas the different precursor sentences had a cross-covariance value of 0.20. To estimate the across-frequency temporal coherence of the sentences, they were split into six octave-wide spectral bands, with center frequencies ranging from 200 to 6400 Hz. The amplitude envelope for each band was calculated using the Hilbert transform and lowpass filtered at 50 Hz. The cross-covariance coefficients were then calculated for all pairwise combinations of the six bands, converted to a *z* score using Fisher's *r*-to-*z* transform and then averaged before converting back to an *r* value. As expected, the cross-covariance coefficient was substantially larger for coherent precursors (0.33) than for incoherent precursors (0.08).

For the experimental data, each participant's answer was evaluated as correct or incorrect, and the proportion of accurate responses was determined for each experimental condition. A generalized linear mixed model for data analysis effectively compared datasets with different distributions while also handling repeated measurements from the same participants (Rabe-Hesketh *et al.*, 2002). The dependent variable was accuracy for each condition, the fixed effects were precursor condition (coherent, incoherent, silence) and which pitch the listeners had to attend to (higher, lower), with a random intercept to accommodate listener variability. The analysis employed a log-link function and a Poisson mixed model (estimated using ML and Nelder-Mead optimizer). Data analysis was performed using R Statistical Software (version 4.3.1; R Core Team, 2023) with the glmer function from the lme4 package (Bates *et al.*, 2015).

In addition to the main analysis, we employed Bayesian methods to investigate the strength of evidence for any null effects observed (Kruschke, 2021; Lakens *et al.*, 2020). To do so, the approach evaluates how well *H*_0_ (the null hypothesis) and *H*_1_ models predict the observed data, and this is quantified as the Bayes factor (*BF*_01_) (Keysers *et al.*, 2020; Wagenmakers *et al.*, 2018). The Bayesian analysis was performed using JASP 0.18 software with their default priors (JASP Team, 2024).

## 3. Results

**Fig. 2.**
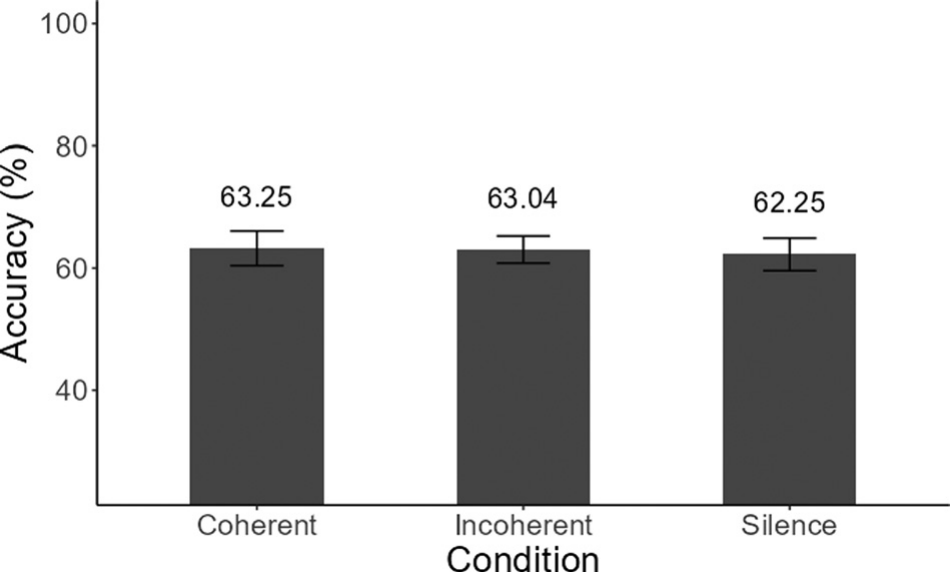
Mean accuracy in reporting the target word under the three different precursor conditions. The presence or coherence of the precursor sentence had no significant effect on listeners' ability to report either the higher or lower target word.

The mean results are shown in Fig. 2. All three conditions resulted in a similar mean level of performance of 62%–63% correct, which was significantly above 50% correct [*t*_(19)_ > 4.58, p < 0.01]. The criterion of 50% correct was chosen, as it corresponds to chance performance if it is assumed that the two words presented on each trial are both heard, but not necessarily attributed to the correct sentence. The Poisson mixed model's total explanatory power was substantial (conditional R^2^ = 0.46), but the part related to the fixed effects alone was 0.01. Neither the fixed factors (precursor type: *χ*^2^ = 0.07, p = 0.97; pitch of target voice: *χ*^2^ = 0.68, p = 0.41), nor their interaction (*χ*^2^ = 0.21, p = 0.90), had a statistically significant effect on participants' accuracy. Bayesian one-way ANOVA was used to examine the likelihood of the null hypothesis *H*_0_, relative to the hypothesis that the precursor type affects performance (*H*_1_). A Bayes factor (*BF*_01_) between 3 and 10 (indicating that *H*_0_ is 3–10 times more likely than *H*_1_) is interpreted as strong evidence for *H*_0_ (Jeffreys, 1998). Our analysis resulted in a *BF*_01_ = 7.17, indicating that the observed data are more than 7 times more likely to occur under the null model than under our predicted model. These results therefore provide strong evidence that listeners' accuracy was not affected by the type of precursor or by the pitch of the attended voice.

**Fig. 3.**
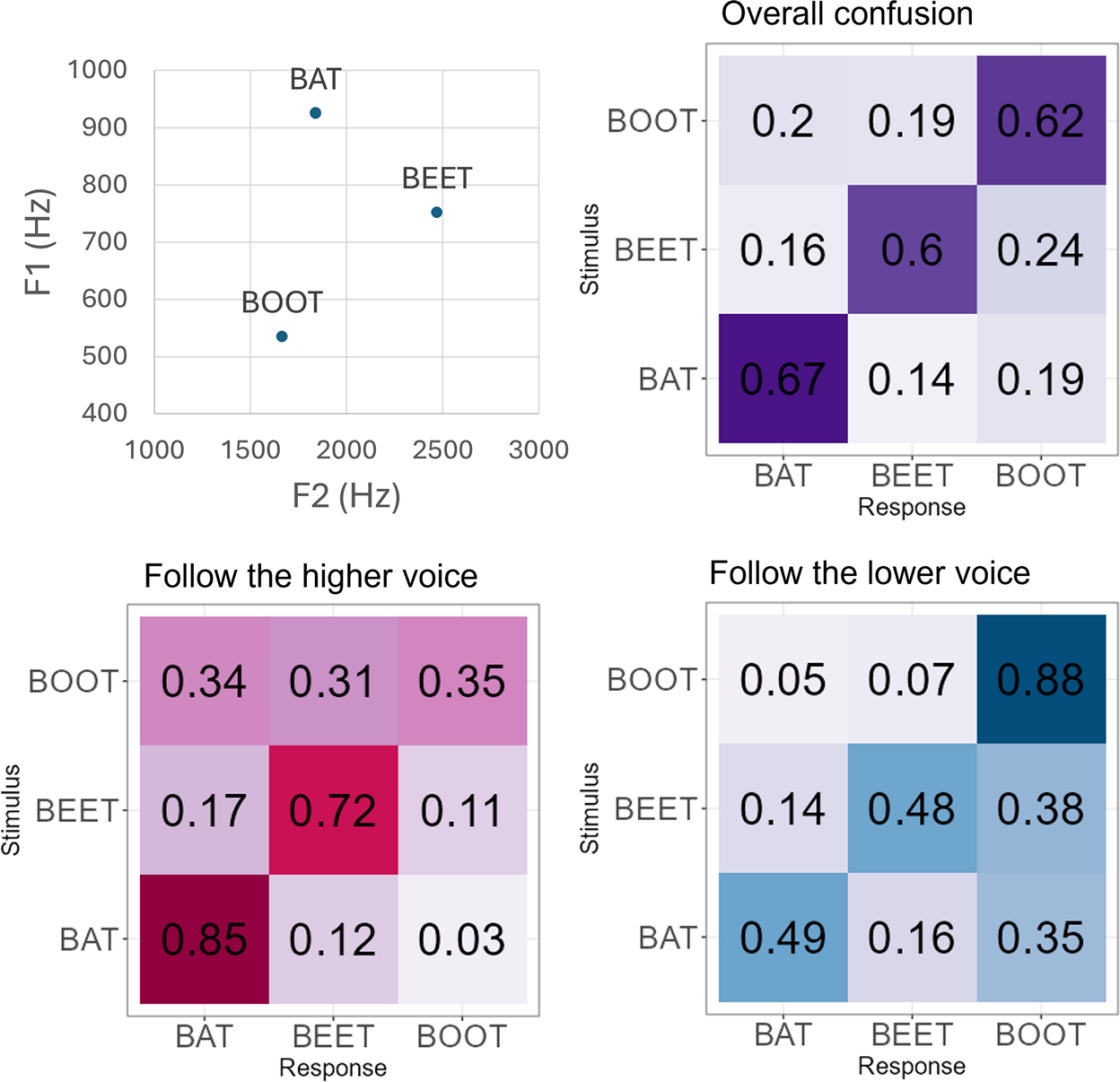
The upper left panel shows the F1–F2 vowel space for the three target words. The upper right panel shows the confusion matrix for responses pooled across all conditions (target voice high or low). Each cell shows the proportion of responses (columns) for each presented stimulus word (rows), with darker shading indicating higher values. The two lower panels show confusion matrices of the same data, but separated by the pitch of the target, with the high target shown in the left panel and the low target shown in the right panel.

Confusion matrices were constructed to determine if there were systematic patterns of responses that depended on whether the low or high voice was attended. There seem to be no systematic trends in the overall pattern of confusion (Fig. 3, top right). However, when subdividing the responses based on the pitch of the target voice, BAT and BEET were more often chosen as the response when the higher voice was the target (Fig. 3, bottom left), whereas listeners were more likely to report BOOT when the lower voice was the target (Fig. 3, bottom right). This pattern of errors likely occurred because vowel sounds like [a] (BAT) or [ē] (BEET) tend to have a higher fundamental frequency than the vowel [ü] (BOOT) (Taylor, 1933; Turner and Verhoeven, 2011), although in our recordings the maximum F0 difference between the vowels (∼1 semitone) was considerably smaller than the 3-semitone difference between the low and high voice. Also, as shown in Fig. 3 (top left), and in line with many previous studies of formant frequencies (Gelfer, 1995; Ryalls and Lieberman, 1982), BOOT has the lowest F1 and BAT and BEET have the highest F1 and F2, respectively, another reason why BOOT may be more likely to be associated with the low voice.

## 4. Discussion

The purpose of this study was to determine whether evidence for perceptual organization based on temporal coherence could be observed in the presence of competing talkers, as it had for simpler psychoacoustic experiments in the past (Elhilali *et al.*, 2009; Krogholt Christiansen and Oxenham, 2014; Sollini *et al.*, 2022). Listeners were asked to follow a target sentence that was either temporally coherent or incoherent with a competing sentence on a different mean F0. The prediction was that the final word of the target sentence should be more easily identifiable if the preceding part of the sentence was accompanied by a temporally incoherent competing sentence, than if it was preceded either by a temporal coherent competing sentence or by silence. Contrary to our prediction, the temporal coherence of the precursor phrases did not have a significant effect on the accuracy of identifying the correct keyword. This outcome suggests that the findings from simpler psychoacoustic experiments on temporal coherence do not necessarily generalize to more complex conditions involving speech.

One possibility is that the single sentences used in our study did not provide sufficient time for perceptual segregation to build up. It is well known that the gradual accumulation of acoustic information can lead to improved perceptual performance in tasks involving segregation (Bregman, 1978; Fritz *et al.*, 2007) and detection of sounds (Hicks and McDermott, 2024; King and Walker, 2020). However, previous studies have typically reported that the time over which buildup occurs is between 0.4 and 2.5 s, depending on the task and stimuli (Brandewie and Zahorik, 2010; Holt, 2006; McDermott *et al.*, 2013). Thus, our sentence duration of about 2 s should have been sufficient to achieve the amount of buildup necessary to observe an effect segregation based on temporal coherence.

Another possibility is that the average F0 difference between the two sentences was too small or too large for performance to be further modulated by changes in temporal coherence. However, based on previous studies, our choice of 3 semitones should have been sufficient to produce performance that was substantially better than that observed with no pitch difference between talkers, but poorer than that observed with a larger pitch difference (e.g., 9 semitones or more) (Başkent and Gaudrain, 2016; Darwin *et al.*, 2003; Madsen *et al.*, 2017). Thus, there should have been scope for an additional benefit of temporal coherence to be observed.

Finally, it is possible that the nature of the task (to report just the last word) changed listeners' strategy to the extent that temporal coherence effects were not observed because listeners were not attending to the preceding portion of the sentence. If so, however, it is not clear why the effects of temporal (in)coherence were observed in psychophysical studies, where ignoring the preceding sequence of stimuli could also have led to improved performance. Nevertheless, it would be informative to revisit the question of the role of temporal coherence in speech using a task that involved longer speech segments and more continuous tracking requirements.

Overall, our current results provide no support for the hypothesis that temporal coherence plays an important role in speech segregation. This conclusion, although surprising, is indeed consistent with the two previous studies that have also explored the effects of temporal coherence on the perceptual segregation of speech stimuli. First, David *et al.* (2018) found no effect of asynchrony between two sequences of consonant-vowel pairs on listeners ability to attend to one and ignore the other, so long as there remained some temporal overlap between the stimuli. Second, Lee and Oxenham (2024) found that disrupting the temporal coherence of a target talker by introducing temporal asynchrony between spectral bands did not result in a greater degradation in performance for a task involving segregation of two talkers than for a task simply involving speech in noise. Finally, as noted by Lee and Oxenham (2024), the across-frequency temporal coherence of speech (0.33 in our case) is considerably lower than the value of 1 (perfect coherence) used in most psychoacoustic studies (e.g., Elhilali *et al.*, 2009; Krogholt Christiansen and Oxenham, 2014). It may be that the perceptual effects of temporal coherence only emerge at these (artificially) high levels of across-frequency temporal coherence.

Our empirical findings contrast with observations from computational models (e.g., CASA frameworks, Conv-TasNet), which prioritize temporal and spectral coherence, underscoring a critical and potentially fruitful gap between human auditory strategies and machine learning approaches. Nevertheless, it remains possible that other behavioral approaches, perhaps using longer passages and different speech-tracking tasks, will have more success in highlighting the role of temporal coherence in the perceptual organization of speech in complex backgrounds.

## Data Availability

The data that support the findings of this study are available from the corresponding author upon reasonable request.
